# Firearm locking device preferences among firearm owners in the USA: a systematic review

**DOI:** 10.1186/s40621-023-00436-7

**Published:** 2023-07-06

**Authors:** Jessica Buck-Atkinson, Megan McCarthy, Ian H. Stanley, Ben Harnke, Michael D. Anestis, Craig. J. Bryan, Justin C. Baker, Marian E. Betz

**Affiliations:** 1 Firearm Injury Prevention Initiative, School of Medicine, University of Colorado Anschutz Medical Campus, 12401 E 17th Avenue B215, Aurora, CO 80045 USA; 2 Injury and Violence Prevention Center, Colorado School of Public Health, University of Colorado Anschutz Medical Campus, 13001 East 17th Place B119, Aurora, CO 80045 USA; 3 Department of Emergency Medicine, School of Medicine, University of Colorado Anschutz Medical Campus, 12401 E 17th Avenue B215, Aurora, CO 80045 USA; 4 Center for COMBAT Research, Department of Emergency Medicine, School of Medicine, University of Colorado Anschutz Medical Campus, 12401 E 17th Avenue B215, Aurora, CO 80045 USA; 5 Strauss Health Sciences Library, University of Colorado Anschutz Medical Campus, 12950 E Montview Blvd, Aurora, CO 80045 USA; 6 Rutgers School of Public Health, New Jersey Gun Violence Research Center, 683 Hoes Lane West, Piscataway, NJ 08854 USA; 7 School of Public Health, The State University of New Jersey, 683 Hoes Lane West, Rutgers, Piscataway, NJ 08854 USA; 8 Department of Psychiatry and Behavioral Health, The Ohio State University College of Medicine, 3650 Olentangy River Rd, Suite 330, Columbus, OH 43214 USA; 9 VA Eastern Colorado Geriatric Research Education and Clinical Center, Denver, CO USA

**Keywords:** Firearms, Secure storage, Locking devices, Guns, Lethal means safety

## Abstract

**Background:**

Preventing firearm-involved injuries is a critical public health priority. Firearm locking devices can prevent firearm injuries, such as suicide and unintentional shootings, as well as theft. Various firearm locking devices exist; however, little is known about firearm owners’ preferred locking devices for secure firearm storage. In this systematic review, we examined existing literature on preferred locking devices for secure storage of personal firearms among United States (US) firearm owners with the purpose of understanding practical implications and needs for future research.

**Methods:**

We searched 8 major databases, as well as the grey literature, for English-language sources published on or before January 24, 2023, that empirically examined firearm locking device preferences. Following PRISMA guidelines, coders independently screened and reviewed 797 sources using pre-determined criteria. Overall, 38 records met inclusion criteria and were included in this review.

**Results:**

The majority of studies measure and report on participant use of various types of locking devices, but few go on to measure preference between device options and the attributes and features that may contribute to an individual’s preference. Included studies suggest that a preference for larger devices, such as lockboxes and gun safes, may exist among US firearm owners.

**Conclusions:**

Review of included studies suggests that current prevention efforts may not be aligned with firearm owners’ preferences. Additionally, findings from this systematic review emphasize the need for additional methodological rigorous research to understand firearm locking device preferences. Expanded knowledge in this area will result in actionable data and foundational best practices for programming that encourages behavior change concerning secure storage of personal firearms to prevent injury and death.

## Background

In the USA, firearm injuries, spanning suicide, homicide, interpersonal violence, and unintentional shootings, are a major public health concern. Each year, over 45,000 individuals in the USA die due to firearm injuries, and tens of thousands more experience nonfatal injuries annually (Aitken et al. 2020; National Center for Injury Prevention and Control 2023; Rees et al. 2022). Multiple studies have demonstrated that when a firearm is stored securely (e.g., locked, unloaded, and separate from ammunition), risk for firearm suicide and other forms of firearm-involved injuries may be reduced (Monuteaux et al. 2019; Shenassa et al. 2004). Thus, to reduce the risk of firearm injuries, key stakeholders—including medical organizations (Butkus et al. 2018; McLean et al. 2019; Bulger et al. 2019), suicide prevention organizations (American Foundation for Suicide Prevention 2022), leaders in the firearm-owning community (National Shooting Sports Foundation 2022a), and other groups—encourage secure firearm storage practices.

An estimated 30% of US adults own one or more firearms and an additional 11% do not personally own a firearm but live with someone who does (Parker et al. 2017). One prominent approach to secure firearm storage is the use of firearm locking devices. While an estimated 36% of US firearm owners store all of their firearms locked (Parker et al. 2017), there are various types of firearm locking devices available to firearm owners, spanning keyed cable locks (which commonly rely on a steel cable) to biometric safes (which use biological data, such as fingerprints, unique to authorized users) (National Shooting Sports Foundation 2021). Prior research examining firearm locking device use has not considered the heterogeneity in locking device preferences.

Promisingly, interventions that distribute firearm locking devices increase end users’ secure firearm storage practices (Anestis et al. 2021d; Roszko et al. 2016). Work to date has largely focused on the distribution of cable locks, a relatively low-cost option, although there may be variations in firearm owners’ preferences for certain types of firearm locking devices (Stuber et al. 2021). Interventions that are attentive to firearm owners’ preferences may have the potential to increase their receptivity to recommendations to store firearms securely.

In this systematic review, we sought to examine published studies on firearm locking device preferences among US firearm owners to help inform public health and clinical decision-making regarding the distribution of firearm locking devices. In addition to examining studies specifically focused on firearm locking device preference, we also included studies examining firearm owners’ current use of specific locking devices (e.g., cable lock vs. gun safe), as one’s current choice to use a device may be a proxy, albeit imperfect, for current preferences. We also sought to identify gaps in the existing literature and present recommendations for future research in this area.

## Methods

### Data sources and search strategy^1^

Our search strategy was developed and implemented by a health sciences librarian (Rethlefsen et al. 2015). Records were eligible for inclusion if they were: (1) published in English; (2) empirically (quantitatively or qualitatively) examining US firearm owners’ firearm locking devices preferences; and (3) peer-reviewed studies, dissertations, and non-peer-reviewed publications/organizational reports or presentations. Studies had to be US-based. No limits on publication date were used. Conference abstracts and proceedings were excluded in the search strategies for databases with high conference proceedings coverage (PsycINFO, Sociological Abstracts, Web of Science). We queried 8 databases with an updated search on January 24, 2023: Ovid MEDLINE, Web of Science, PsycINFO, Public Affairs Information Service Index, Sociological Abstracts, Social Sciences Full Text, ProQuest Dissertations & Theses A&I, and Google Scholar (first 100 citations). The title, abstract, and subject headings for select databases (Ovid MEDLINE, PsycINFO) were searched for key vocabulary. See Table 1 for full search strategy and terms for Ovid MEDLINE.

**Table 1:** Search strategy

Database(s): Ovid MEDLINE(R) ALL		
#	Searches	Results
1	((firearm* or handgun* or gun or guns or pistol* or rifle* or shotgun* or weapon*) and (store or storing or stored or storage or lockup or lock or locks or locked or locking or lockbox* or (safe* adj2 device*))).tw,kf. or (Firearms/ and Protective Devices/)	1047
2	(interview* or theme* or qualitative or attitude* or perspective* or perception* or survey* or questionnaire* or opinion* or prefer* or behavior or behavior or behaviors or behaviors or belief* or plan or planning or planned or plans or focus group*).tw,kf. or exp qualitative research/ or focus groups/ or interviews as topic/ or “surveys and questionnaires”/ or Narration/	4,408,084
3	1 and 2	375
4	limit 3 to English language	370
5	remove duplicates from 4	370

Additionally, we conducted a search for grey literature via custom, advanced Google searches developed using similar strategies. Our grey literature search included targeted searches of the organizational websites listed in Table 2, as well as several search strings, which were iteratively defined and developed by the review team.

**Table 2:** Grey literature organizations searched

Organization	URL
Pew Research	www.pewresearch.org
National Opinion Research Center	www.norc.org
SSRS	www.ssrs.com
Gallup	www.news.gallup.com
Johns Hopkins	www.jhsph.edu
Giffords	www.giffords.org
Roper Center	www.ropercenter.cornell.edu
Small Arms Survey	www.smallarmssurvey.org
American Foundation for Suicide Prevention	www.afsp.org
National Shooting Sports Foundation	www.nssf.org
The Educational Fund to Stop Gun Violence	www.efsgv.org
Safer Homes, Suicide Aware	www.saferhomescoalition.org
Defense Suicide Prevention Office	www.dspo.mil
Firearm Safety Among Children and Teens	www.icpsr.umich.edu
Bulletpoints	www.bulletpointsproject.org

### Study selection

Study selection methods and procedures followed PRISMA guidelines. The initial database search yielded 1316 citations. Records were de-duplicated for identical citations and organized using the citation management software Endnote version 20 (Clarivate). After de-duplication, the remaining 797 records were uploaded to Covidence, a systematic review citation screening software, which identified three additional duplicates—leaving 794 for title and abstract screening.

**Fig. 1:**
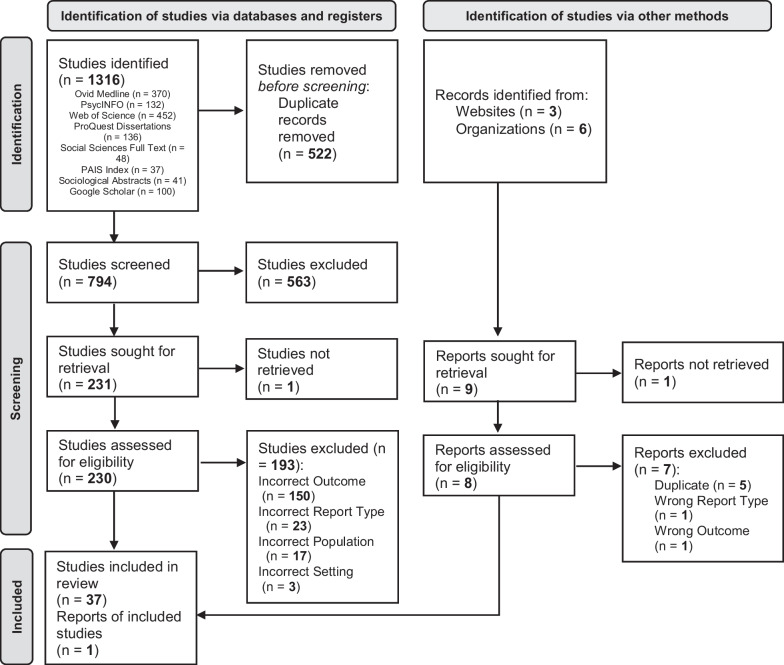
PRISMA diagram

Three reviewers—a trained Research Assistant with a Bachelor of Public Health and two trained undergraduate student supervisees—independently screened the titles and abstracts of all 794 records. The reviewers were supervised by senior members of the Review Team, including the Project Manager and the Principal Investigator who both have extensive experience in firearm injury prevention research and clinical and public health programmatic efforts. Following screening, a full-text review of the 231 remaining records was conducted by the same three reviewers. Each record was independently reviewed by at least two reviewers. Any discrepancies across reviewers were discussed with the larger Review Team and resolved with consensus. Following the full-text review, 37 records met inclusion criteria. The search of the grey literature resulted in the inclusion of one additional record that met eligibility criteria. Eligibility of grey literature was determined first by a title screen, followed by a full-text review conducted by two independent reviewers. Upon conclusion of the full-text review and grey literature search, 38 records were eligible for data collection (Fig. 1).

### Data collection and quality assessment

The Review Team collaborated to develop and refine a data extraction form within Covidence. Two team members—the Project Manager and the Research Assistant—independently extracted the following data from each included record: citation, year, location, study setting, participant characteristics, study aims, methodology, and reported outcomes related to firearm locking device use, preference, and willingness to pay for devices. Data collection was conducted using Covidence. Any disagreements between the reviewers were resolved by consensus of the team members.

To determine if the identified studies were of sufficient methodological quality, we used the Critical Appraisal Skills Programme (CASP), a tool frequently used to assess the quality, utility, and relevance of studies (CASP 2023). Using this 10-item metric, the Review Team methodically examined each included article across various study domains (clear methodological aims and approach, appropriate research design and participants, data collection and interpretation, relevance, and utility of findings), providing a “Yes,” “No,” or “Can’t Tell” response to each item. All Review Team members used a structured methodological approach for assessing selected articles with this tool. Disagreements in assessment were resolved via discussion between reviewers and it was determined that only studies scoring a 7/10 or higher were to be included. All 38 studies were deemed to be of sufficient methodological quality and therefore met the threshold to be included.

### Data synthesis and analysis

Due to the extent of differences across included studies in methodology, design, and outcomes, no meta-analyses were feasible. Instead, the study team chose to report findings as a narrative synthesis, summarizing and explaining the characteristics and results of the included studies pertaining to the primary outcomes of this review.

## Results

We identified a total of 38 studies that reported on firearm locking device preferences among US firearm owners.^2^ Included articles were published from 1996 to 2022. Two included studies examined the same dataset. Sample sizes ranged from 16 to 6404. Table 3 provides a list of all included studies, their reported aims, sample size, participants, setting, methods, types of firearm locking devices discussed, and whether devices were provided. In this section, we summarize the findings in the literature regarding (1) preferences for specific firearm locking devices; (2) device attributes and features contributing to preferences; (3) current use of specific firearm locking devices among firearm owners; and (4) firearm owners’ willingness to pay for devices.

**Table 3:** Included Records (n = 38)

Citation	Study aim(s)	Sample size	Participants	Setting	Methods*	Type(s) of devices**	Devices provided
Aitken et al. (2020)	Better understand parental knowledge, attitudes, beliefs, and firearm storage practices that will support interventions to lower unsupervised child access to firearms	57	Own any type of firearm; children < 18 years in the home	Alabama, Arkansas, Tennessee	BOTH: Focus groups; in-person survey	Gun safe, trigger lock	Yes, trigger locks
Anestis et al. (2021a)	Determine differences in preferred messengers on the topic of safe firearm storage and suicide prevention between firearm owners and non-firearm owners, and among firearm owners of different racial groups and sexes.	6200 total 2311 firearm(s) in or around the home	$\ge$18 years	U.S.	QUANT: Web-based Survey	Trigger lock, cable lock, gun safe, gun cabinet, lock box	No
Anestis et al. (2021b)	Assess frequency of suicidal ideation among individuals who purchased firearms during the surge period, other firearm owners, and non–firearm owners	6404 1546 current firearm owner	$\ge$18 years	Mississippi, Minnesota, and New Jersey	QUANT: Web-based survey	Gun safe, cable lock, trigger lock	No
Anestis et al. (2021c)	Determine the extent to which COVID-19 firearm purchasers differ in terms of suicide risk from non-firearm owners and firearm owners who did not make a purchase during COVID-19	3500 1164 firearm owner	$\ge$18 years	Web-based U.S.	QUANT: Web-based survey	Gun safe, gun cabinet	No
Anestis and Houtsma (2019)	Understand factors that differentiate firearm owners’ beliefs and behaviors that are relevant to suicide risk by examining differences between individuals for whom firearm ownership represents a central aspect of identity (i.e., primary firearm owners) versus firearm owners who primarily identify with some other demographic or occupational characteristic.	375	Firearm owner	U.S.	QUANT: Web-based survey	Gun safe; trigger lock, cable lock	No
Barber et al. (2022)	Describe safety standards for locking devices and compare parental acceptance rates for different types of devices	226 85 firearm(s) in the home	Parent of child (10–17 years) at emergency department (ED) for a suicide-related or behavioral health-related problem	Emergency departments in Colorado	QUANT: Phone and web-based survey	Cable lock, lock box	Yes, cable lock or lock box
Barton and Kologi (2015)	Expand knowledge of factors surrounding firearm storage practices including individual storage practices, personal experience with firearms, and views on firearms ownership	30 24 firearm owner or in home	$\ge$18 years	Small town in Pacific Northwest	BOTH: Interview; in-person survey	Trigger lock, gun safe, lock box, gun cabinet, “gun lock” unspecified	No
Carbone et al. (2005)	Evaluate the effectiveness of gun-safety counseling, a gun-safety brochure, and a free gun lock in subsequent gun removal and safe storage	151	Parent of patient < 18 years; gun owner	Pediatric clinic within community health center in Tucson, AZ	QUANT: Phone survey	Lock box, “gun lock” unspecified	Yes, cable lock
Coyne-Beasley et al. (2005)	Examine the level of agreement on household firearms and storage practices among cohabiting partners	76 partner-pair respondents	Previously received hospital-based intervention; $\ge$18 years; with child for care; children < 18 years in home; speak English	North Carolina	QUANT: Phone survey	Trigger lock, lock box, gun safe, gun cabinet; “gun lock” unspecified	No
Coyne-Beasley et al. (2001b)	Determine if a firearm safety counseling and gun lock distribution program improved storage practices	112	Event participant	Community-based firearm safety event in urban county in North Carolina	BOTH: In-person survey; interview	Lock box, gun safe, gun cabinet, “gun lock” unspecified	Yes, cable locks and raffled gun safe
Coyne-Beasley et al. (2001a)	Examine law enforcement officers’ willingness to use gun locks on their own guns, as well as their opinions regarding gun locks in general	75	Agency officer with service weapon	Law enforcement agency in Southern region of the U.S.	QUANT: survey	Cable lock	Yes, cable locks
Crifasi et al. (2019)	Estimate the desirability of personalized guns among a nationally representative sample of current gun owners	1444	Gun owner	U.S.	QUANT: Web-based survey	Biometric	No
Crifasi et al. (2018)	Examine gun storage practices and factors influencing those practices among gun owners	1444	Gun owner; $\ge$18 years	U.S.	QUANT: Web-based survey	Gun safe, gun cabinet, lock box, trigger lock	No
Dennis et al. (2019)	Conduct assessment for the Portland-metro area regarding preferences for gun storage devices	147	Current/soon-to-be caregiver of child; current or future gun owner	Community events and hospital lobby in Portland, OR	QUANT: In-person surveys	Cable lock, life jacket, lock box, gun safe, biometric	No
DeMello et al. (2020)	Assess parent acceptance of firearms education delivered by clinical providers, determine whether parents engage in firearms safety dialog with their children, and evaluate reasons for ownership and storage behaviors	115 47 reported owning firearms	Parent and/or guardian of patient	Pediatric inpatient surgery center in Houston, Texas	QUANT: In-person survey	Trigger lock, cable lock, lock box, gun safe	No
Denno et al. (1996)	Ascertain the specific suggestions that local police departments in the USA give to parents who ask for advice about methods to safely store handguns	93 law enforcement agencies	Police department in city with population $\ge$10k	U.S. cities	QUANT: Phone survey	Lock box, trigger lock	No
Furman et al. (2020)	Evaluate the impact of a mobile safety center on pediatric home safety knowledge and device use	50 11 reporting firearm(s) in the home at baseline	$\ge$18 years; parent or guardian of children < 18 years in home	Community-based events in Pennsylvania	QUANT: In-person survey	Gun safe, “gun lock” (unspecified)	Yes, “gun lock”
Grossman et al. (2012)	Determine if the installation of gun cabinets improved household firearm storage practices	255 households	$\ge 21$ years; principal owner or renter of dwelling; 1 + gun usually present in home; do not possess operational gun safe	Households in 6 rural villages in Bristol Bay and Yukon-Kuskokwim Delta regions of western Alaska	QUANT: In-person survey	Gun safe, gun cabinet, cable lock, trigger lock	Yes, gun cabinets
Grossman et al. (2000)	Determine the effectiveness of gun safety counseling during well-child care visits	1292 households 309 reporting owning firearms at baseline	Scheduled visit for a child 2 months to 18 years	Primary care clinics in Washington state	QUANT: In-person survey	Trigger lock, lock box	Coupons for obtaining a trigger lock and a lock box at a discount
Horn et al. (2003)	Develop and evaluate a pilot program to reduce unauthorized access to firearms by youth by distributing gun safes and trigger locks to households	40 households	Village resident; $\ge$18 years; homeowner or primary renter; owner of 2 + long guns; no gun safe	Households in two villages in Southwest Alaska	QUANT: In-person survey	Gun safe, trigger lock	Yes, gun safes and trigger locks
King et al. (2020)	Describe characteristics of community-based firearm safety event participants and assess whether presence and age of children in the household were associated with household firearm locking practices	2956 2480 reported firearm(s) in the home	$\ge$18 years; speak English or Spanish	Community-based firearm safety events in Washington state	QUANT: In-person survey	Gun safe; lock box; cable lock; trigger lock	Yes, lock box or trigger lock
Kuhls et al. (2017)	Evaluate Committee on Trauma member attitudes about firearm ownership, freedom, responsibility, physician-patient freedom and policy	237 101 reported firearm(s) in the home	U.S. American College of Surgeons Committee on Trauma members	U.S.	QUANT: Web-based survey	Gun safe, trigger lock	No
Lam (2019)	Determine receptiveness and responsiveness in promoting lock box and trigger lock giveaway events on social media and describe characteristics of participants who find out about the event through social media	414 social media comments; 4509 survey participants	Interaction with event pages (content analysis); event participants receiving a trigger lock or lock box (survey)	Community-based firearm safety events in Washington state	BOTH: Social media content analysis; in-person survey	Gun safe, gun lock, trigger lock, cable lock	Yes, trigger lock or lock box
Monteith et al. (2020)	Explore female veterans’ firearm-related experiences and perspectives	16 14 reported currently owning firearms	Female veteran; 20–70 years; reported current/previous gun owner or lives in home with gun(s); eligible to receive Veterans Health Administration care in the Mountain West; no severe psychiatric symptoms or cognitive impairment	VA Medical Center in Mountain West	QUAL: Interview	Gun safe; lock box	No
Ramchand et al. (2018)	Explore the degree of past exposure to violence among recent suicide cases, characteristics of ownership and storage practices among those who used a firearm to take their lives, and insight from those with access to firearms who chose to end their lives another way	17	Next of kin of suicide cases	New Orleans, LA	QUAL: In-person or phone Interviews	Lock box, trigger lock	No
Roberto et al. (2002)	Assess the effectiveness of a radio-based health communication intervention promoting trigger lock use	237	$\ge$18 years; called to receive a trigger lock in response to hearing radio PSA	Michigan	QUANT: Mail-based survey	Trigger lock	Yes, trigger locks
Schenck et al. (2022)	Characterize parental attitudes and beliefs related to firearm storage and identify facilitators and barriers to safer storage practices	20	Parents/guardian of a child < 18 years; live in home with firearm(s)	Pediatric clinics and ED in New Haven County Connecticut	BOTH: In-person or telephone interview and survey	Lock box	Yes, cable lock
Schuster et al. (2000)	Determine the prevalence and storage patterns of firearms in US homes with children	6990	Reported children in the household < 18 years	U.S.	QUANT: In-person survey	Trigger lock	No
Sidman et al. (2005)	Examine a multifaceted community education campaign to promote safe handgun storage and the campaign’s impact on firearm locking and loading practices in households with children	151 households	Speak English; 1 + child < 18 years in home; telephone in the home; 1 + pistol, revolver, or other handgun kept in/around home	Western region of the U.S.	QUANT: Phone survey	Trigger lock, gun safe, lock box	Yes, coupons for lock boxes
Simonetti (2018b)	Conduct a preliminary evaluation of a community-based firearm safety intervention and assess participants’ preferences for firearm locking devices and their comfort with potential firearm safety counselors	206 191 firearm(s) present in the home	$\ge$18 years; speak English or Spanish	Community-based firearm safety events in King and Pierce counties, Washington State	QUANT: In-person survey	Cable lock, trigger lock, lock box, gun safe	Yes, lock box or trigger lock
Simonetti (2018a)	Describe firearm storage practices and correlates of those practices among a nationally-representative sample of U.S. Veteran firearm owners	561	Veteran; report personally owning gun in working order	U.S.	QUANT: Web-based survey	Trigger lock, cable lock, gun safe, lock box	No
Simonetti et al. (2019)	Describe preferences for firearm locking devices and device features among firearm safety event participants	401	$\ge$18 years; speak English or Spanish	Community-based firearm safety events in Washington state	QUANT: In-person survey	Trigger lock, cable lock, Life Jacket, lock box, gun safe	Yes, lock box or trigger lock
Sullivant et al. (2022)	Test whether a brief community-based presentation that includes an emphasis on safe storage, paired with the tools needed to enact safe storage of firearms and medications, can lead to adoption of safe storage practices	581 220 firearm(s) present in home	English-speaking adult; raising children at least half of the time	Midwest Region	QUANT: Web-based survey	Cable lock	Yes, cable lock
Uspal et al. (2021)	Determine if providing firearm storage devices with training during clinical care improves safe storage practices in household members of children who present to a pediatric hospital with an emergent mental health complaint	164	Proficient in English; store 1 + gun in home shared with patient < 18 years	Emergency department or inpatient psychiatric unit within a pediatric hospital in Washington state	QUANT: In-person, phone, and e-mail surveys	Trigger lock, lock box	Yes, lock box or trigger lock
Walters et al. (2012)	Examine Veteran, family member, clinician and VA leaders’ perceptions and ideas regarding gun accessibility and safety among VA patients	60	Mental health diagnosis; active VA mental health care; current/ previous access to guns in the prior 5 years	VA Medical Center in Midwest	QUAL: Interviews; focus groups	Trigger lock, lock box	No
Wargo et al. (2013)	Change gun safety behavior through safer storage of firearms in the home	58	Head Start family; children aged$\le 6$	Head Start intake meetings in central Pennsylvania	QUANT: In-person and phone surveys	Gun cabinet	Yes, “gun lock”
Webb (2022)	Survey families in ED about safety behaviors before and after provision of free safety devices related to drowning, poisoning, and firearms	357 161 firearm(s) present in home	Care-giver with relation to patient present in the ED; English-speaking	Large, urban ED in Alabama	QUANT: In-person and phone surveys	“Trigger lock (cable gun lock with key)”	Yes, “trigger lock (cable gun lock with key)”
Wexler et al. (2022)	Describe and understand people’s associations with and actions related to firearms, safety, and storage in rural Alaska Native communities	33	Alaska Native adult from community	Communities in rural Alaska	QUAL: In-person focus groups	Gun safe, trigger lock	No

*QUAL qualitative methods were used, *QUANT* quantitative methods were used, and *BOTH* both quantitative and qualitative methods were used

**Locking devices discussed or reported

### What preferences for firearm locking devices exist among firearm owners in the USA?

Eight studies reported data that suggest which firearm locking devices firearm owners may prefer. In three studies, participants were offered a free locking device and allowed to pick between lockboxes and trigger locks. Lockboxes were chosen by more participants in each study compared to trigger locks (82–18% of firearm owners Uspal et al. 2021; 87–12% of all participants Simonetti et al. 2018b; 89–8.5% of firearm owners King et al. 2020), two of these studies—both with at least 90% of sample reporting firearm ownership—also reported less than 2% of all participants having “no preference” between the options (Simonetti et al. 2018b; King et al. 2020). Barber et al. (2022) also reported on participant selection and eventual use of either cable locks or lock boxes. Among the sample of parents whose child (10–17) was being evaluated in an emergency department for a suicide-related or behavioral health-related problem, fewer opted to receive offered cable locks (65%) compared to offered lockboxes (70%). A similar pattern emerged at follow-up, with more firearm-owning participants reporting use of the provided lock box (28%) compared to use of the provided cable locks (14%).

A study of 1292 families with children ages 2 to 18 years measured the use of coupons provided to participants to purchase subsidized devices (Grossman et al. 2000). Intervention participants who reported owning firearms received a coupon to purchase a lock box (median redemption price $9.99, range $9.99–$45.00; average retail price ~$70.00) and one to purchase a trigger lock (median redemption price $0.00, range $0.00–$5.00, average retail price ~$10.00). More coupons were used to purchase lock boxes (8.4%, n = 26/309) than trigger locks (4.9%, n = 15/309). In a study of 401 community-based firearm safety event attendees, Simonetti et al. (2019) found that a greater proportion of firearm-owning participants indicated they would never use a trigger lock, cable lock, or clamshell device compared with a lock box or gun safe. These studies may suggest a preference for larger. More expensive devices (e.g., safes and lockboxes) compared with cable and trigger locks, which are smaller in size and often cheaper in cost.

### What attributes or features contribute to firearm owners’ preferences for various types of locking devices?

Ten studies reported on the device features and/or attributes that may influence firearm owners’ preference for and use of various devices. Several of these studies collected information from participants not on reasons for one device versus another, but rather on overarching features and attributes influencing the use of any locking device. These findings are consistent with previous research on motivations to use locking devices (Thomas et al. 2022; Hamilton et al. 2018; Crifasi et al. 2018) and emphasize the barriers to using a variety of locking devices among firearm owners who choose to own firearms for self and household protection (Warner 2022; Cao et al. 1997; Schenck et al. 2022). One study conducted with 147 firearm-owning parents and child caregivers reported 75% of participants indicated both the speed of being able to unlock and lock a device and being able to keep the firearm loaded when locked as “absolutely essential” features (Dennis et al. 2019). Simonetti et al. (2019) found that over 80% of community-based firearm safety event attendees with firearms in their homes reported the same features to be “very important” or “absolutely important”. This was supported by several additional studies that employed qualitative methodologies to collect information from participants, reporting hesitancy to use lock boxes and trigger locks due to delayed access in the event of a home invasion. Schenck et al. (2022) quoted one participant, “If someone’s in your house, you have literally seconds before they’re right there in your face. So, you have to find the key, get to the box, then you got to get to the ammo, unlock it, put it all together, I’m already dead at that point.”

No studies reported on the *use* of biometric devices, but one study of 1444 firearm owners reported on participants’ hesitancy to use biometric devices in place of more traditional devices. Biometric devices are commonly suggested as a solution to quick access concerns, but concerns noted in this study included vulnerability to hacking and the potential that the technology would fail or malfunction when needed (Crifasi et al. 2019). Cost was also discussed across several studies as a barrier to use of larger devices (e.g., gun safes) and/or biometric devices.

A study of 75 law enforcement officers with issued firearms reported on unfavorable attributes specific to cable locks that include a key. Officers expressed worry about losing the key and damage to or deterioration of the key mechanism (Coyne-Beasley and Johnson 2001a). A study conducted with 40 Alaskan firearm-owning households reported unfavorable features of trigger locks, with the most common reason for not using trigger locks being that they were “inconvenient” (27% of participants) (Horn et al. 2003). Several studies also reported motivating attributes including a device’s ability to be used for both handguns and long guns, ease of transfer (e.g., between vehicle and home), and ease of installation and use.

### What firearm locking devices are used by firearm owners in the US?

Most (76.3%; 29/38) studies reported on participant use of locking devices at the time of study involvement, which we included because one’s current choice to use a device may be a proxy for current preferences. Across studies, the firearm locking devices reported on varied, as did the labels and descriptions used to define devices. Table 3 lists these locking device categories measured in each study.

There were notable differences across studies in study design, procedures, measures, participant inclusion criteria, and sample size. To facilitate comparisons with cross-sectional observational studies, we focused on the baseline proportions of firearm locking device use reported for studies that involved an intervention and/or a follow-up component in this review. Most studies (86.2%; 25/29) collected information on locking device use via self-report surveys. Eleven studies collected survey data from general populations of adults, with sample sizes ranging from 30 to 6,404. The use of gun safes was the most reported, with representation in nine survey studies of general adult populations, followed by lock boxes (n = 7), trigger locks (n = 6), cable locks (n = 4), and gun cabinets (n = 3). Only one study reported on the use of clamshell devices. No studies reported on the use of in-vehicle locks or biometric devices. Studies that reported on current device use only among firearm owning participants, showed the following ranges of use by device type: cable lock 18.7–29.2%, trigger lock 16.3–21.4%, gun safe 25.5–52%, and lockbox 6–20.1%.

Nine survey studies required participants to be parents or guardians of or to live in a household with children under the age of 18. Sample sizes across these studies varied (range 50−6990) and included both firearm owners and non-firearm owners, as did the devices included. The devices most asked about (4 studies) included gun safes (reported use ranging from 14.8 to 54.5%) and trigger locks (9–48.5%). The use of lockboxes (three studies: 9.2–48.8%), cable locks (two studies: 11% and 16.8%) and gun cabinets (two studies: 19.7–28%) were measured less often. Two studies included multiple devices in the same response option (e.g., safe or trigger lock), making it unclear which device participants were actually using (Aitken et al. 2020; Carbone et al. 2005).

### What are firearm owners willing to pay for various firearm locking devices?

Six studies explored the cost of firearm locking devices and the role cost played in participants’ use of various devices. One study conducted with firearm-owning parents and caregivers of children under the age of 18 found that gun safes were often seen as too expensive to independently buy and use (Aitken et al. 2020; DeMello et al. 2020). In Simonetti et al.’s (2019) study of 401 community-based firearm safety event attendees, participants reported how important various features of locking devices were to them. 22% of firearm-owning participants felt device cost being less than $15 was “very important” or “absolutely essential”. Finally, a survey among 147 Oregon-based current and soon-to-be parents explored which devices firearm owners would prefer if “money was not an issue”. Options included a cable lock, life jacket locking device, lockbox with keyed access, quick access electronic lockbox, and biometric lockbox. 54% of respondents selected a biometric device as their first choice and 20% selected a gun lockbox with electronic keypad access. The majority of participants reported a gun lockbox with electronic keypad access as their second choice. Cable locks were reported overall as the least favorable choice (Dennis et al. 2019).

Included studies also explored the provision of free and/or subsidized devices. One study of 164 parents or caregivers of pediatric patients reported that participants were more likely to accept a locking device that was free, compared with a locking device that was available at a reduced cost (91% vs. 52%) (Uspal et al. 2021). Overall, 12 included studies provided at least one type of free locking device: Six provided trigger locks, four cable locks, three lock boxes, and three gun safes or cabinets. One additional study provided free devices but did not specify which device type was given.

## Discussion

Reducing firearm-involved injury and death will take a multilayered, community-engaged approach. In recent years, there has been increasingly more outreach to and leadership from firearm owners and the firearm industry to promote secure firearm storage (National Shooting Sports Foundation 2022b). Expansion of efforts to understand locking device preferences will provide practitioners, policymakers, and other stakeholders with useful insight on how to design effective safety interventions.

Review of the included studies suggests that current prevention efforts that employ the provision of cable and trigger locks—locking devices that are generally smaller and less expensive—may not be aligned with what firearm owners prefer. In fact, the review of reported outcomes indicates that a preference for larger devices, such as lockboxes and gun safes, may exist. Feasibility and scalability require a balanced consideration of cost and preference to ensure optimal implementation of interventions. In this case, a clearer understanding is needed regarding the proportion of firearm owners that would adopt secure firearm practices if their preferred—but more expensive—storage devices were made readily available. Such work would be useful in determining the degree to which preference findings should influence device distribution strategies. Future research might consider using customer-value-based pricing questionnaires (Garrison and Towse 2017) that enable an understanding of the price point at which the cost of specific firearm storage devices influences the likelihood that firearm owners would purchase and use specific devices. Such information could help establish not only a sense of the market for specific devices but could also enable cost-benefit analyses that aid in determining what types of devices specific outreach programs might opt to offer. Additionally, it is important to note that preference may be influenced more so by locking mechanism (e.g., key, combination lock, etc.) than by size and price, but the included studies did not include information regarding mechanism.

Our focus on preference for specific locking devices builds upon the growing body of research that seeks to understand motivations and barriers to the practice of storing and staging firearms locked. One theme that aligned with previous research is the prominence of firearms being kept unlocked to increase the speed of access in case of self-defense (Warner 2022; Cao et al. 1997). Individuals who are motivated to own firearms for self and home protection reportedly see locking devices in opposition to this motivation. Indeed, included studies reported participants using locking devices on some personally owned firearms while always leaving one or more unlocked. A possible solution may be biometric devices, which allow for quick access and prohibit unauthorized access and use. However, this systematic review revealed that little is known about firearm owners’ preferences for biometric devices over traditional devices. In fact, the few and limited studies that explored biometric devices with participants reported features and attributes that may make them undesirable for firearm owners, although additional research is needed. As research in this area moves forward, it will be important to understand the potential intersection between reason for firearm ownership (e.g., self- or home-defense) and specific firearm locking device preferences. To be clear, the findings from this systematic review are not dispositive regarding which firearm owners prefer which firearm locking devices and the associated characteristics thereof. Until more representative research can be conducted, practitioners may consider offering multiple device options for free or at a reduced cost.

There exists a variety of factors that may influence an individual firearm owner’s preferences for firearm locking devices (Hamilton et al. 2018; Crifasi et al. 2018; Ramchand 2022). These factors include motivations for ownership, number and type of firearms owned, household makeup, neighborhood characteristics, and more. Interventions that acknowledge the breadth of individual factors by allowing owners to choose their preferred device may be the most effective.

## Limitations and future directions: extant literature

Limitations of the extant literature include the limited number of studies and extent of differences in methodology, design, and outcomes did not allow for an analysis past a narrative synthesis. Because of this, our ability to compare the results of one study to another or to draw detailed conclusions that are likely to reflect the bulk of US firearm owners is severely limited. Future research can address this in multiple ways. First, efforts should be made to recruit large, representative samples of specific communities of firearm owners, allowing the research to accurately reflect the diverse array of firearm-owning communities (Thomas et al. 2022) and to highlight any differences that may emerge regarding storage preferences. This type of nuanced and generalizable understanding would facilitate optimization of resource distribution. Second, the research community should develop preferred standards for assessment methods that enable easier comparison of results and future meta-analytic consideration of these questions. Like any area in which limited research has been conducted, it is unsurprising to find substantial variation in how questions are framed and what information is assessed, but as the field advances, more consistent operationalization will be vital. Third, we were also unable to assess the extent to which device preferences vary by type of firearm owned (e.g., handgun vs. long gun) and reason for ownership. It may be that individuals have varying preferences based upon the degree to which they desire quick easy access (e.g., for a home protection firearm vs. one used primarily for hunting). This information will provide enhanced recommendations for practitioners and policymakers pertaining to the most effective approaches to device provision as part of lethal means safety effort. If, for instance, evidence emerges demonstrating that preferences for specific locking devices vary based on the extent to which firearm owners more readily envision themselves using the firearm to fend off an intruder than someone using it for another reason more likely to cause harm to the firearm owner or other household residents (e.g., suicide, unintentional shootings), this would speak to the need for increasing awareness about the actual risks for specific firearm-related outcomes in the home, particularly if further data indicate specific locking devices are more effective at preventing such outcomes. Fourth, it is vital that researchers systematically collect data on storage preferences across diverse samples, thereby clarifying if and how locking device preferences differ across demographics (e.g., race, gender, parenting status, geographic location). Fifth, additional research should examine the extent to which firearm owners’ current firearm storage practices align with their preferences for specific firearm locking devices and identify reasons for potential discrepancies. Sixth, going forward researchers should assess whether preferences for locking devices differ between firearm owners who do and do not currently use locking devices and between firearm owners who lock all their firearms relative to those who keep at least one firearm unlocked. The existence of any such differences is not clear based on the current literature and, in fact, no such differences may emerge following direct assessment; however, efforts to promote the use of locking devices focus specifically on those who do not currently lock their firearms, so understanding variation in preferences will be vital to the success of such campaigns.

## Limitations: current systematic review

There are also limitations of the current systematic review that are important to note. First, we only included studies that were published in English; although the focus was on the USA, it remains possible that non-English speaking reports exist. Second, although our search was broad—across 8 databases—and conducted in consultation with a health sciences librarian, we did not examine every possible database; thus, it is possible that some studies may have been missed by this search strategy. Third, conference abstracts and proceedings were excluded in the search strategy from a subset of the databases, potentially missing studies that have not been submitted to or which were triaged in peer-reviewed journals. Additionally, although we developed our search terms collaboratively within a team of researchers with extensive experience in this area, it remains possible that we chose suboptimal terms and, because of this, our search did not reveal other instances of extant relevant research. Finally, we did not comprehensively examine industry and consumer data (e.g., purchasing patterns), which might provide critical insights into firearm locking device preferences. An additional important consideration is that our discussion of firearm storage practices—a variable related to but not synonymous with firearm storage preferences—is not based on a systematic review of the firearm storage practices literature. Our findings are restricted to results presented in studies that also report explicitly on storage preferences and, as such, numerous other studies that report firearm storage practices are not represented in our findings. Future efforts may be successful in accessing this information if partnerships with the firearm industry are cultivated and nationwide purchasing data are made more readily available for research purposes.

## Conclusions

This systematic review provides important information and identifies knowledge gaps for future work. The findings from 38 total studies provide an initial summary of what data have previously been collected from firearm owners—including that firearm owners may prefer lock boxes or safes to cable locks, and that cost and access (speed and reliability) are concerns. More importantly, this review emphasizes the need for additional research to understand the topic and improve firearm-involved injury prevention efforts that involve the provision of free or reduced-cost locking devices. Until additional research can be conducted, practitioners should provide multiple device options for free or at a reduced cost to firearm-owning individuals and communities, as this may address the various factors that influence an individual’s decision to own firearms and, therefore, their preference for which locking device(s) to use on their weapon(s).

## Data Availability

The datasets used and/or analyzed during the current study are available from the corresponding author on reasonable request.

## Footnotes

1 The protocol for this systematic review is registered with PROSPERO and available at https://www.crd.york.ac.uk/prospero/display_record.php?ID=CRD42022309531.

2 The contributing author MA is also the lead author on four studies included in this review. MA contributed to the preparation and review of this manuscript but was not involved in screening and review therefore limiting bias.
